# Determinants of recovery time from severe acute malnutrition among cholera-exposed and unexposed children in Ethiopia: a prospective cohort study

**DOI:** 10.3389/fnut.2024.1463150

**Published:** 2024-10-03

**Authors:** Alemayehu Belay Alamneh, Kalkidan Hassen Abate, Ashagre Molla Assaye, Yeshambel Worku Demlie, Moti Edosa Guma, Tefera Belachew

**Affiliations:** ^1^Public Health Emergency Management, Ethiopian Public Health Institute, Addis Ababa, Ethiopia; ^2^Department of Human Nutrition & Dietetics, Institute of Health, Jimma University, Jimma, Ethiopia; ^3^Department of Nursing, College of Medicine and Health Sciences, Bahir Dar University, Bahir Dar, Ethiopia

**Keywords:** children, cholera, Ethiopia, malnutrition, nutrition, severe acute malnutrition, time to recovery

## Abstract

**Background:**

There is a vicious interplay between severe acute malnutrition (SAM) and diarrheal diseases including cholera. The lack of sufficient evidence on the time to recovery and its determinants among children with cholera prompted this investigation. The study aimed to evaluate the time to recovery and determinants among children with severe acute malnutrition, comparing cholera-exposed and unexposed children.

**Methods:**

A prospective cohort study was carried out from September 10, 2022, to February 21, 2023, among 224 children below 15 years. The study was conducted at cholera treatment centers and nearby communities in the Bale and Guji Zones of Oromia Regional state in Ethiopia. A structured questionnaire was used to obtain information and anthropometric measurements were conducted weekly. After checking all assumptions, a multivariable Cox Proportional Hazards model was used to identify independent determinants of time to recovery using an adjusted hazard ratio (AHR) with a 95% confidence interval (CI). Statistical significance was declared at *p* < 0.05.

**Results:**

Nearly 80% of participants recovered from SAM with a recovery rate of 40/1000 person-week observation and a median time to recovery of 21 days [Inter Quartile Range14-28]. The recovery time from SAM for cholera-exposed children was delayed by 54% [ARR: 0.46, 95% CI: 0.30–0.69] compared to unexposed children. Similarly, the recovery time from SAM for food-insecured families was delayed by 39% [ARR: 0.61, 95% CI: 0.38–0.96] compared to food-secured families. Moreover, children with more than three meal frequencies per day had 1.61 times [ARR: 1.61, 95% CI: 1.04–2.50] higher probability of fast recovery from SAM, compared to children with less than three meals. Children from families with good attitudes toward nutrition had more than two times [ARR: 2.23, 95% CI: 1.45–3.41] higher probability of faster recovery from SAM than families with poor attitudes.

**Conclusion:**

The study revealed that cholera exposure is one of the main determinants of prolonged recovery time for children with SAM. Food insecurity, meal frequency, and the attitude of parents toward children’s dietary habits were determinants for the recovery of children from SAM. The findings imply the need for designing interventions to enhance child feeding during cholera illness, focusing on key determinants.

## Introduction

Childhood malnutrition continues to be a significant global health issue, resulting in illness, death, and disability. It encompasses various nutritional disorders, such as underweight, wasting, stunting, and micronutrient deficiencies. Wasting specifically refers to acute malnutrition caused by insufficient recent intake of necessary nutrients, which can be influenced by diarrhea and other acute illnesses (1, 2).

The world’s children are facing an epidemic of malnutrition. In 2022, the 15 most severely affected countries account for over 30 million children suffering from wasting, a condition characterized by a significant loss of weight and muscle mass. Disturbingly, it has been observed that a mere 5 % increase in the actual cost of food elevates, the global risk of wasting by nearly 9 %. This highlights the vulnerability of children to the economic fluctuations affecting the affordability and accessibility of nutritious food (3).

Moreover, an estimated 45 million children under 5 years old were suffering from wasting worldwide. In 2024, the convergence of threats may further increase the number of children, and pregnant and breastfeeding women affected by acute malnutrition (4).

On the other hand, about 150 million under 5 years children are estimated to be too short for their age or stunted, and about 50 million are too thin for their height or wasted, most of the world’s wasted children are living in South Asia and Sub-Saharan Africa (5). And account for about 50% of direct or indirect causes of global under-five mortality (6).

After years of grappling with undernutrition, Africa is now facing a concerning rise in overnutrition that is characterized by excessive intake of calories, leading to obesity and associated health problems. The rise in obesity rates can be partially attributed to changes in modern lifestyles. These lifestyle shifts include increased consumption of processed and high-calorie foods, as well as more sedentary behaviors and reduced physical activity (7). Despite the surge in overnutrition and the double burden of undernutrition remains an important public health problem in many African countries requiring a multidisciplinary for its solution (8). Populations in South Sudan, Somalia, and Ethiopia are experiencing starvation and concurrent outbreaks of cholera besides this, the combination of drought, conflict, and population displacement in certain countries has led to a significant increase in food insecurity and a higher prevalence of severe acute malnutrition (SAM) among the affected populations (9).

According to the United Nations Children’s Fund (UNICEF), World Health Organization, and World Bank Group joint malnutrition estimate in 2018, about 14 million wasted children lived in Africa, and 4 million were specifically found in East Africa (10). Like many countries in sub-Saharan Africa (SSA), Ethiopia has a high burden of malnutrition among children with 36.6% stunted, 12.2% wasted, and 25.2% underweight (11).

Children with acute malnutrition (AM) have a higher incidence and longer duration of diarrhea due to mainly electrolyte imbalance, which in turn affects the treatment of dehydration, especially in severely malnourished children. The burden of acute malnutrition among children in Ethiopia also remains high, which might have an impact on treatment outcomes (12). Evidence showed that there is an intersection of cholera with malnutrition (12). However, data on the two together and the treatment outcomes among children having cholera and acute malnutrition are limited in Ethiopia. A study conducted in the Lay Armachiho district of Ethiopia found that children with poorly diversified diets and inadequate micronutrient intake are at a higher risk of impaired growth and development. This research highlights the prevalence of undernutrition among schoolchildren in a region where evidence has been limited (13).

The recovery time of children with SAM is influenced by age, dietary diversity, micronutrient intake, weight, vaccination status, poverty level, education, occupation, household food insecurity, and comorbidities such as pneumonia, stunting, shock, and deworming (7, 13–16). Household characteristics such as poverty level, education, occupation, household food insecurity, and dietary diversity are associated with subsequent wasting and stunting in children (16). Effective management of SAM with cholera remains a huge challenge in low-resource healthcare settings (17). Moreover, comorbidities were the main determinant factors; SAM children with severe wasting, pneumonia, vomiting, and anemia had prolonged stays in the stabilization centers without recovery (18).

Although there are guidelines and protocols for treating children with both cholera and SAM, specific research on their relationship is limited. For example, the Global Task Force on Cholera Control (GTFCC) provides detailed protocols for managing children with SAM during cholera outbreaks, emphasizing careful rehydration and monitoring to prevent complications such as over-hydration by overlooking nutritional intervention. However, most existing literature addresses the broader implications of infectious diseases on malnutrition without thoroughly examining cholera’s unique impact on recovery time from SAM (19).

Previous studies conducted in Ethiopia (5, 14, 20–23) focused mainly on SAM and did not address the comorbidity of SAM with Cholera, and its impact on the recovery rate. This makes the effort to treat children with cholera and improve their recovery and survival rate very challenging. Thus, the need to study the comorbidity of SAM and cholera became evident and this study was conducted to fill this gap. Therefore, the study aimed to identify the time to recovery and its determinants among children with severe acute malnutrition with cholera and without.

## Methods

### Study design and setting

This prospective cohort study was conducted to examine the time to recovery and determinants influencing time to recovery among both cholera-exposed and unexposed children under the age of 15 years. Prospective cohort studies offer valuable insights into disease prevention and intervention effects on malnutrition. However, challenges arise when it comes to following participants, especially in cases of dropout or loss of follow-up. To ensure the robustness and reliability of our findings, we employ rigorous methods and address potential sources of bias.

The study was conducted at cholera treatment centers and nearby communities in the Bale and Guji Zones of Oromia, Ethiopia. The capital town of Bale, Robe, is located 402 kilometers away from Addis Ababa (Southwest direction). Bule hora, the capital of Guji zone is located 477 kilometers away from the capital city, Addis Ababa. The data were collected from September 10, 2022, to February 21, 2023. The cholera treatment centers (CTCs) were located in cholera hotspot districts in Ethiopia, as identified by the Ethiopian Public Health Institute (24). There were 14 CTC centers in Oromia Regional State during the study period. The study areas were selected due to the cholera outbreak occurrence during the study period.

### Study population and sampling

The study population comprised two groups: cholera-exposed children with SAM under the age of 15 and cholera-unexposed children with SAM of the same age. The cholera-unexposed children with SAM were identified by screening the nutritional status of children from the nearby respective communities where the CTCs are located. The stratified random sampling method was used to select the participants exposed to cholera. The cholera hotspot areas served as the strata, and two cholera treatment centers (CTCs) in the Guji Zone and 12 CTCs in the Bale Zone were selected for inclusion in the study. Simultaneously, strata were identified in nearby communities for the control group, and participants were randomly selected from each stratum. For the unexposed group, strata were created based on the kebele where the cholera treatment center was established and the study participants were selected using a simple random sampling technique using the list of households as a sampling frame from the nearest kebele (strata) to the cholera treatment center.

### Sample size determination

The sample size was determined using Epi StatCalc version 7.2.4 software, where P1 is the prevalence of wasting among unexposed children in Nepal, is 12.6% (25), and P2 is the prevalence of acute malnutrition among exposed children with cholera in Nigeria, which is 24% (12). With the power (P) = 90%, and the confidence level of 95%, by adding 10% for non-response, the overall sample size was 566 [550 with a 97.1% response rate], for those children undergoing SAM screening. A 1:1 (275:275) ratio for the exposed and unexposed groups was considered. Then, those who developed SAM from both groups were followed for this particular time to recovery study.

During the screening for severe acute malnutrition (SAM), 224 children in both study groups were identified as having a mid-upper arm circumference (MUAC) measurement below 11.5 cm. The screening was conducted at the CTCs for cholera exposed and house-to-house screening was conducted for non-exposed children. These 224 children with MUAC <11.5 cm were considered for this follow-up study.

### Inclusion and exclusion criteria

The study included children under the age of 15 with SAM who were both exposed and unexposed to cholera. Additionally, only SAM children who were found from both groups were included in the final sample for follow-up. Both the exposed and unexposed groups of children with no parents or delegated caregivers, as well as with known chronic diseases, were excluded from the study. Cholera cases were confirmed by experts from the Ethiopian Public Health Institute.

### Data collection techniques and tools

A structured questionnaire was developed in English and translated into local languages (Amharic and Afaan Oromo) for better understanding by the participants. To ensure consistency, the translated questionnaire was back-translated into English. The questionnaire was modified and adapted from Ethiopian protocols for the management of severe acute malnutrition as well as from various literature sources (26–32). The data collection team consisted of 10 professionals. Eight of them were data collectors who held Bachelor’s degrees, and two were supervisors with Master’s degrees.

The principal investigator provided training to the data collectors and supervisors, covering the study’s objectives, the measurement techniques of each characteristic, completing the questionnaire, and addressing any issues encountered. Data were collected from cholera-exposed and unexposed children using the Open Data Kit (ODK) data collection tool and follow-up registration log book.

A well-structured questionnaire (Supplementary 1) was used in the data collection process that involved obtaining information by interviewing parents of cholera-exposed children at the time of their discharge from the CTC. MUAC measurements were taken from children during their follow-up time. Concurrently, data were gathered from the unexposed group within the community. The data collection procedure entailed conducting face-to-face interviews with parents or caregivers and measuring the mid-upper arm circumference (MUAC) of the sample children.

The study had two groups of participants (cholera-exposed and unexposed children), and the data were collected using an Open Data Kit (ODK) application. The data collection process involved obtaining information from the parents of cholera-exposed children at the time of their discharge from the cholera treatment center. Simultaneously, data were gathered from the unexposed group within the respective nearby strata in the community. The mid-upper arm circumference (MUAC) was measured. Severe acute malnutrition (SAM) was determined using a MUAC measurement with a cutoff value of less than 11.5 cm based on the Ethiopian SAM guidelines. From both Cholera-Exposed and Unexposed groups, children with MUAC measurement <11.5 cm undergo the follow-up to determine the time to recovery and the factors associated. Further definitions of key terms are included in Supplementary 2.

During data collection, children with a mid-upper arm circumference (MUAC) measurement less than 11.5 cm were identified as having severe acute malnutrition, regardless of whether they were in the exposed or unexposed groups. These participants with severe acute malnutrition were then included in the analysis.

The follow-up of SAM children was monitored weekly at health posts, health centers, and their homes for 6 weeks. Follow-up was done at health centers until SAM children were able to eat and move independently after which, they were linked to health posts for further follow-up. The SAM Children who can eat and move independently were followed at health posts that directly came from the health center, the CTC, or traced from the community using the SAM screening program weekly. All the SAM children were checked every week starting from the date of screening and diagnosis for SAM, and the event status was determined every week (outcome of interest or censored). Additionally, the study ensured regular monitoring of the children every week during the follow-up period, which helped to minimize the potential delays in determining the outcome of interest.

### Data analysis

The main outcome variable is time to recover from SAM among children. The data were imported from the Open Data Kit (ODK) tool to SPSS and Stata for cleaning and analysis. Statistical Package for Social Science (SPSS) version 25 software was used for descriptive analysis and to generate lifetable. Most statistical analyses were done using STATA version 18 including generating a cumulative hazard plot of Cox-Snell residuals for model fitness and the Kaplan–Meier survival curve.

The outcome for each participant was dichotomized into either censored or event/recovered. Children whose mid-upper arm circumference (MUAC) measurement was greater than 11.5 cm were considered to have recovered. Participants who missed two or more consecutive weekly follow-up visits were classified as defaulters. Those children who defaulted (missed two or more consecutive weekly visits) or died during the study period were categorized as censored observations.

Life table analysis was employed, to estimate the cumulative proportion of survival time at different time points. The Kaplan–Meier test was used, to compare the median recovery time between various independent groups. The log-rank test was also used to examine the significant relationship between the time to recovery and the different independent groups. These analytical techniques were used to comprehensively understand the survival and recovery patterns within the study population and the potential influence of various independent determinants on the time-to-recovery.

To check the proportionality of hazards among different groups of individuals, the Proportional Hazards (PH) assumption was employed. This assessment was conducted subjectively using Kaplan–Meier estimators, predicted survival curves, and log–log plots of survival curves. In addition, an objective evaluation was performed using Schoenfeld residuals.

The analysis sought to identify variables that demonstrated a good fit within the model. Variables that met this criterion demonstrated parallel lines in the survival graphic presentation and yielded a global test statistic with a *p*-value greater than 0.05. These variables were considered to adhere to the PH assumption and included in the model. Finally, the Cox–Proportional hazard model was used after checking all the assumptions of the PH model. Variables with a *p*-value less than 0.25 in the bivariable analysis were selected and included in the multivariable analysis. A multivariable Cox proportional hazards model was fitted to control the confounding variables. The statistical analysis with a *p*-value less than 0.05 was declared as a statistically significant association.

## Results

### Background characteristics of children with SAM

**Figure 1:**
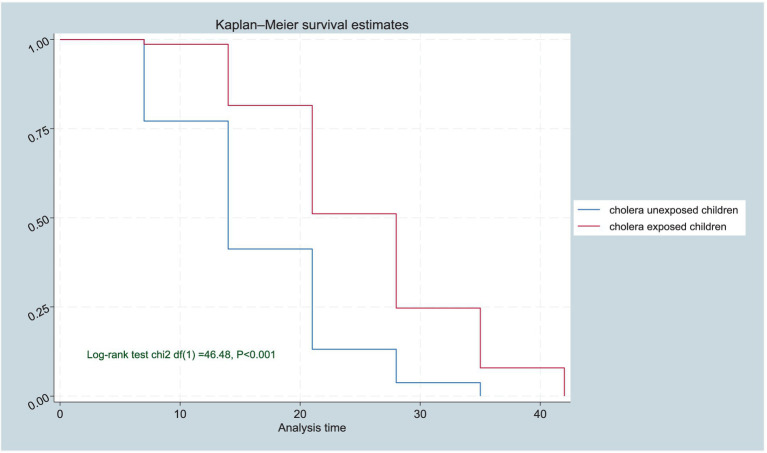
Kaplan–Meier survival function estimates of cholera-exposed and unexposed SAM children, in Bale and Guji Zones of the Oromia Region, Ethiopia from 20 September 2022 to 21 February 2023 (*n* = 224).

In this study, a total of 550 children were screened for SAM with a 97.1% response rate. From the screened children, 224 children with SAM were included. Half of the screened children (275) were cholera-exposed. The children’s mean (±SD) age was 3.6 (± 2) years. Among the participants 121 (54%) of the children were males. Of them, 158 (70.5%) children were under 5 years old. Three-fourths of the children 167 (74.6%) came from parents with more than four family members. A high percentage of parents, 202 (90.2%) had no formal education. Of the children included in the cohort study 178 (79.5%) [95% CL: 73.6–84.6] recovered from severe acute malnutrition (Table 1; Figure 1).

**Table 1:** Profile of independent variables on time to recovery among SAM children, at Bale and Guji Zones of Oromia Region, Ethiopia from 20 September 2022 to 21 February 2023 (*n* = 224).

Variables		Frequency; n (%)	Cholera exposure status	
			Yes, n (%)	No, n (%)
Sex of children	Male	121 (54)	88 (72.7)	33 (27.3)
	Female	103 (46)	65 (63.1)	38 (36.9)
Age of children	< 5	158 (70.5)	110 (69.6)	48 (30.4)
	5–14	66 (29.5)	43 (65.2)	23 (34.8)
Family size	≤ 4	57 (25.4)	41 (71.9)	16 (28.1)
	>4	167 (74.6)	112 (67.1)	55 (32.9)
Educational status of Parents	Did not Attend school	202 (90.2)	142 (70.3)	60 (29.7)
	Attend school	22 (9.8)	11 (50)	11 (50)
Husband involvement in child-feeding	No	152 (67.9)	99 (65.1)	53 (43.9)
	Yes	72 (32.1)	54 (75)	18 (25)
Food insecurity	Food security	48 (21.4)	43 (89.6)	5 (10.4)
	Food insecurity	176 (78.6)	110 (62.5)	66 (37.5)
Dietary diversity score	Poor	194 (86.6)	127 (65.5)	67 (34.5)
	Good	30 (13.4)	26 (86.7)	4 (13.3)
Food reference of child	Forced to eat	188 (83.9)	134 (71.3)	54 (28.7)
	Change the food	36 (16.1)	19 (52.8)	17 (47.2)
Meal frequency	<3 times	166 (74.1)	108 (65.1)	58 (34.9)
	≤ 3 times	58 (25.9)	45 (77.6)	13 (22.4)
Water source	None pipe	171 (76.3)	103 (60.2)	68 (39.8)
	Pipe	53 (23.7)	50 (94.3)	3 (5.7)
Water treatment practice	No	122 (54.5)	69 (56.6)	53 (43.4)
	Yes	102 (45.5)	84 (82.4)	18 (17.6)
Water treatment type	Traditional	74 (33)	69 (93.2)	5 (6.8)
	Chemical	28 (12.5)	15 (53.6)	13 (46.4)
Knowledge of nutrition	Poor	125 (55.8)	92 (73.6)	33 (26.4)
	Good	99 (44.2)	61 (61.6)	38 (38.4)
Attitude on nutrition	Poor	91 (40.6)	70 (76.9)	21 (23.1)
	Good	133 (59.4)	83 (62.4)	50 (37.6)
Oral Cholera vaccine status	Not fully Vaccinated	195 (87.1)	139 (71.3)	56 (28.7)
	fully Vaccinated	29 (12.9)	14 (48.3)	15 (51.7)
Recovery status	Recovered	178 (79.5)	120 (78.4)	58 (21.6)
	Censored	46 (20.5)	33 (81.7)	13 (18.3)

In the study, there are many independent variables and their profiles are recorded below in the table. That includes 176 (78.6%) of children who came from parents who suffered from food insecurity, and almost three-fourths, 166 (74.1%) of them had less than three times the frequency of meals per day. In addition, 194 (86.6%) of them had poor dietary diversity. Even though 133 (59.4%) parents had a good attitude toward nutrition, only 72 (32.1%) of the husbands were involved in child-feeding practices. On the other hand, 125 (55.8%) of parents had poor nutrition knowledge. Regarding food preference, 188 (83.9%) of children’s food preferences were not kept rather they were forced to eat available food in the house (Table 1).

Most of the parents, 171 (76.3%) relied on unprotected water sources such as rivers, wells, or natural springs to meet their water needs. Only, 102 (45.5%) households employ any water treatment method to ensure the safety of their water supply. Of these households, 28 (12.5%) used chemicals for water treatment to make it safe for consumption. Regarding immunization practice, 29 (12.9%) of the children had received full vaccination against cholera (Table 1).

### Time to recover from SAM and the recovery rate

In this cohort study more than three-fourths of children had recovered from SAM with a recovery rate of 40/1000 [95% Cl: 35/1000–46/1000] person-week observation, and with a median recovery time of 21 [95% Cl: 19–22, and IQR: 14–28] days.

The survival table shows that the cumulative probability of not recovering from severe acute malnutrition (SAM) decreased over time. On the 7th day, the cumulative probability of not recovering was 91%. By the 14th day, this probability decreased to 69%. On the 21st day, the cumulative probability was reduced to 38%, indicating that a significant proportion of individuals recovered from SAM at this point. As the follow-up continued, the cumulative probability of not recovering decreased even further. By the 28th day, only 17% of individuals did not recover from SAM in the follow-up. On the 35th day, this probability reduced to 5%, indicating that most individuals had successfully recovered from SAM at this time (Table 2).

**Table 2:** Life table of cholera-exposed and unexposed severe acute malnourished children, in Bale and Guji Zones of the Oromia Region, Ethiopia from 20 September 2022 to 21 February 2023 (*n* = 224).

Interval start time	Number entering interval	Number withdrawing during interval	Number exposed to risk	Number of terminal events	Proportion terminating	Proportion surviving	Cumulative proportion surviving at end of interval
0	224	4	222	0	0.00	1.00	1.00
7	220	21	209.5	18	0.09	0.91	0.91
14	181	5	178.5	44	0.25	0.75	0.69
21	132	11	126.5	56	0.44	0.56	0.38
28	65	3	63.5	35	0.55	0.45	0.17
35	27	2	26	19	0.73	0.27	0.05
42	6	0	6	6	1.00	0	0

### Kaplan–Meier hazard curve and survival difference

**Figure 2:**
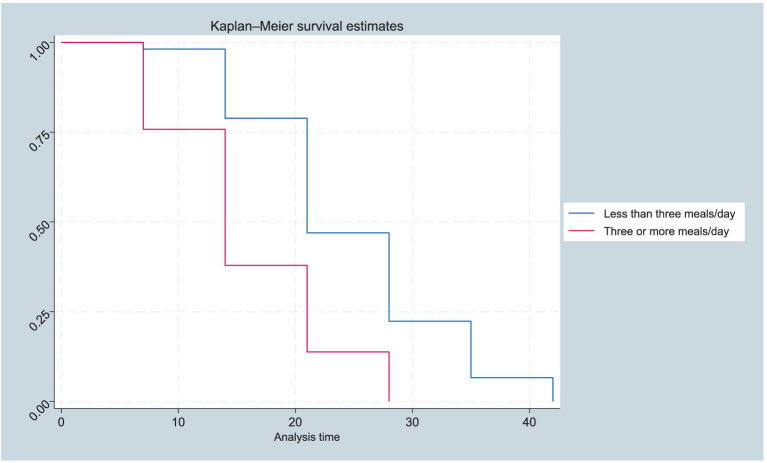
Kaplan–Meier survival function estimates by meal frequency of SAM children, in Bale and Guji Zones of the Oromia Region, Ethiopia from 20 September 2022 to 21 February 2023 (*n* = 224).

The Kaplan–Meier curve analysis provides further insights into the survival and recovery rates of children with Severe Acute Malnutrition (SAM) over time. The results show a significant survival difference in the time of recovery from SAM for cholera-exposed children compared to unexposed children. The generated Kaplan–Meier curves for the two groups (exposed and unexposed) and the log-rank test to determine the statistical significance of the observed difference in recovery rates, evidenced by the log-rank test (*p* < 0·001; Figures 1, 2).

### Determinants of time to recovery among SAM children

The final model allowed for the simultaneous consideration of various determinants and their respective hazard ratios (HRs) while adjusting for potential confounding variables. In the study, the identified determinants include cholera exposure status, household food insecurity, meal frequency, and parent’s attitudes toward nutrition. Accordingly, cholera-exposed children had 54% [ARR: 0.46, 95% CI: 0.30–0.69] less likely to recover quickly from SAM than unexposed children (Table 3).

**Table 3:** Bivariable and multivariable Cox regression analysis of determinants of cholera exposed and unexposed SAM children, at Bale and Guji zones of Oromia Region, Ethiopia from 10 September 2022 to 21 February 2023 (*n* = 224).

Variable		Cholera exposure		CHR [95%CI]	AHR [95%CI]
		Yes (%)	No (%)		
Food security status	Secure	43 (89.6)	5 (10.4)	1	1
	Insecure	110 (62.5)	66 (37.5)	0.76 [0.53–1.09]	0.61 [0.38–0.96]*
Attitude of parents	Poor	70 (76.9)	21 (23.1)	1	1
	Good	83 (62.4)	50 (37.6)	3.17 [2.21–4.54]***	2.23 [1.45–3.41]***
Meal frequency/day	< 3 times feed	108 (65.1)	58 (34.9)	1	1
	≥ 3 times feed	45 (77.6)	13 (22.4)	2.75 [1.87–4.04]***	1.61 [1.04–2.50]*
Cholera exposure status	Un exposed	71 (31.7)	0	1	1
	Exposed	153 (68.3)	0	0.41 [0.29–0.56]***	0.46 [0.30–0.69]***

NB: 1: Reference category; *p-value < 0.05; **p-value < 0.01; ***p-value < 0.001. 2: CHR, Crude Hazard Ratio; AHR, Adjusted Hazard Ratio. ^1^The model was adjusted for the following 10 independent variables, age, Family size, educational status of parents’ cholera exposure status, Food security status, Attitude of parents on nutrition, Meal frequency/day, OCV status, water treatment practice, and food preference of children.

Children from food-insecure families had a 39% [ARR: 0.61, 95% CI: 0.38–0.96] lower probability of fast recovery from SAM than children from food-secure families. In addition, the probability of faster recovery from SAM was 1.61 times higher among children who had three or more meals per day [ARR: 1.61, 95% CI: 1.04–2.50] compared to those who had less than three meals per day. Moreover, children having families with good attitudes toward nutrition had a 2.23 times lower probability of faster recovery [ARR: 2.23, 95% CI: 1.45–3.41] from acute malnutrition compared to their counterparts (Table 3).

**Figure 3:**
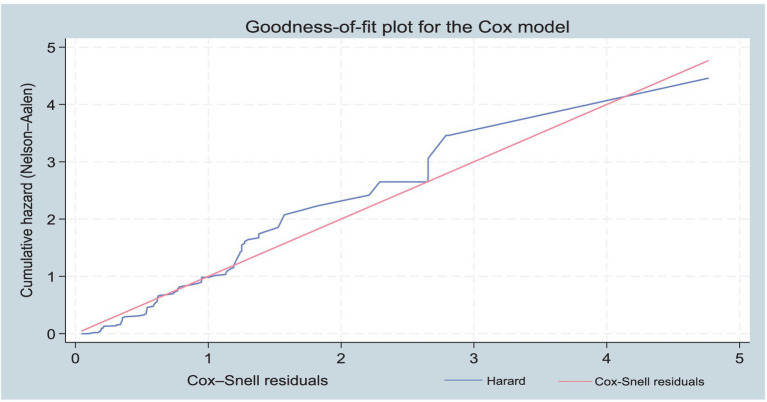
A cumulative hazard plot of Cox-Snell residuals for model fitness.

After conducting the multivariable Cox Regression analysis, the overall Schoenfeld global test was applied to test the assumptions of proportional hazards objectively. Finally, to examine the overall fit of a Cox model, we applied the Cox-Snell plot as if the hazard function followed the 45-degree line. It revealed that the hazard function approximately has an exponential distribution with a hazard rate of one that indicates the model fits the data well (Figures 1, 3).

## Discussion

The study findings indicate that the recovery rate was 79.5% [95% Cl: 73.6–84.6%]. This indicates that even though this finding falls within the acceptable international sphere standards (33), a significant proportion of children (one in five children) did not recover from SAM. This finding is alarming and shows further actions are required to increase the recovery rate of children with SAM. Consistent findings were shown in the study done in Addis Ababa and Southern Ethiopia (14, 23, 34), On the other hand, this study has a lower recovery rate than the study done in Eastern, Ethiopia (35). The possible reason for this variation might be due to Cholera exposure in our study. Cholera exposure can indeed impact recovery rates, as it is a serious bacterial infection that can lead to dehydration and other complications, and a lower recovery rate observed in the population might be linked to increased cholera exposure. In the study, cholera exposure appears to be one of the determinants that contribute to the lower recovery rate observed in the current study population.

The study showed the median time to recover from severe acute malnutrition was 21 days (IQR: 14 to 28 days). This median recovery time, and therefore the length of stay at the stabilization centers, was consistent with the accepted international minimum standard of (<30 days) for the management of severe acute malnutrition (33). Consistent with our study, the research conducted at Jimma University, and Bahir Dar Northwest Ethiopia findings reported a similar time to recovery of severe acute malnutrition in children (28, 29).

On the other hand, the studies conducted in Tigray region, Harar, Yekatit 12 Hospital, Hawassa University, Jimma University, and Woldia Hospital in Ethiopia were found to be a lower time to recovery from SAM than the median recovery time observed in the current study (14, 22, 36–40).

The possible reason for the longer recovery time from SAM in this study may be that it was conducted during an emergency in which most of the participants were exposed to cholera, supply interruptions, and the quality of healthcare provided was called into question. These contextual factors likely contributed to the longer recovery times observed in this study compared to others. In addition, the severity of the included cases is also another factor, when severe cases would inherently require longer recovery periods compared with mild presentations of acute malnutrition, cholera exposure leads to the severity of cases.

Moreover, various studies conducted in the Borena zone of Ethiopia, Southern Ethiopia, and North Gondar zone, Northwest Ethiopia found a longer recovery time than the current study (21, 23, 34). The observed differences in median recovery time across these studies may be due to various factors including the study’s setting, location, and sampling methods. For instance, one study was carried out in the Borena zone within the Oromia region of Ethiopia (23), which is a frequently drought-affected peripheral part of the country. In another study conducted in southern Ethiopia (34), the threshold used to identify children with severe acute malnutrition (SAM) was 11 cm, which captured more severe cases compared to the usual MUAC cutoff point of 11.5 cm. The third study was facility-based (21), exposing participants to additional health conditions that extended the recovery period from SAM.

In this study, the main identified determinants include cholera exposure status, food security status, meal frequency, and parents’ attitudes toward nutrition. The study showed that cholera-exposed children were more than 60% times less likely to recover quickly from SAM. Consistently, various studies have shown that diarrheal diseases, such as cholera exposure, serve as significant determinants of SAM in children (12, 41–44). Diarrheal diseases can exacerbate nutrient deficiencies and wasting, making children more vulnerable to the severe consequences of SAM that make the recovery time longer. Reducing cholera exposure in children and other diarrheal diseases may be an effective strategy for reducing such life-threatening forms of malnutrition in children.

Food security encompasses the availability, accessibility, and utilization of food that meets individuals’ dietary needs and preferences (45). In the context of this study, food security played a crucial role in the recovery process of children affected by severe acute malnutrition. Children from food-insecure families were more than 40% less likely to recover quickly from severe acute malnutrition (SAM) compared to children from food-secure families. Consistent with this finding, other studies conducted in Ethiopia have reported that children from food-secure families had shorter recovery times from SAM than those from food-insecure families (39, 46, 47). Additionally, another study found that an increase in monthly household income was associated with a reduced hazard of recovery from SAM in children (48).

In this study, children who had three or more meals per day were more than one and a half folds more likely to recover from acute malnutrition quickly compared to children who had less than three meals per day. Similarly, other studies have revealed that higher meal frequencies are associated with a lower risk of disease, and inadequate meal frequency is linked to a higher risk of prolonged recovery time from acute malnutrition in children (26, 27, 52).

In contrast to this, the research conducted in South Africa reported that meal frequency is not a reliable predictor of the time to recover from acute malnutrition in children (26). The reason for inconsistency findings may be attributed to differences in study settings which could introduce different variables and influences that impact the recovery process and types of food they eat. This study was conducted in a community-based setting, while the study in South Africa was conducted in a hospital setting, among children receiving quality medical care in a clinical environment, which may have contributed to a lower recovery time observed in that study.

Children from families with positive attitudes toward nutrition increased the recovery rate from severe acute malnutrition (SAM), compared to their counterparts. Similarly, a study conducted in central Ethiopia, found that fathers with positive attitudes toward nutrition were involved in child-feeding practices, compared to those with negative attitudes, studies done among Ugandan caretakers showed that those who had a positive attitude had absolute adherence to nutrition care (49–51). These findings underscore the significant impact that family attitudes and beliefs about nutrition can have on child health and feeding behaviors. Families with more positive, proactive stances toward nutrition appear to provide an environment that facilitates better recovery from malnutrition in children. The findings imply the need for enhanced clinical vigilance and follow-up of the children with SAM exposed to cholera to fasten their recovery and prevent associated mortality and prolonged stay in the health facility.

In the study area, communities frequently move to find food for their cattle. This continuous movement often resulted in missed follow-up appointments, as they would only return home in the evening. Due to this interruption, MUAC measurements could not be taken from the SAM children during their follow-up, leading to a higher rate of defaulters.

## Conclusion

The study revealed that nearly two-thirds of the children recovered from SAM within 3 weeks. The study also showed that cholera exposure is one of the main determinants of prolonged recovery time SAM among children. Various studies had a shorter median recovery time compared to the results of this particular study because of cholera exposure status. Besides cholera exposure status, this study identified key determinants that influence the time to recovery from SAM in children, including, food insecurity, meal frequency, and the attitude of parents toward nutrition.

Healthcare providers and policymakers should take into account the complex interplay between different health conditions when designing interventions to improve recovery outcomes for individuals affected by severe acute malnutrition, especially in contexts where infectious diseases such as cholera pose additional challenges.

Targeted interventions to prevent or mitigate cholera exposure could potentially help improve recovery times among children. Additionally, food insecurity is a key determinant that can influence time to recovery from SAM and nutritional outcomes. Ensuring access to adequate, nutritious food is foundational to combating malnutrition. This emphasizes the importance of implementing interventions that target the underlying factors contributing to food insecurity, such as poverty and agricultural challenges.

Furthermore, adequate and frequent meals are crucial in the fight against malnutrition. Providing children with the necessary caloric and nutrient intake through regular, well-balanced meals can significantly contribute to their recovery and long-term health. Increasing community attitudes about balanced diets is also an essential intervention strategy to reduce malnutrition among children. Educating families on the importance of proper nutrition, feeding practices, and food preparation can empower them to make informed decisions and sustain positive changes in their children’s diets. Developing interventions that account for this multifaceted reality could be more effective in improving recovery times and outcomes for children suffering from SAM, including cholera exposure, food security, meal frequency, and attitudes toward nutrition.

## Data Availability

The materials and data utilized in this study can be accessed upon request to the corresponding author with the approval of the Ethiopian Public Health Institute.

## References

[1] Bizuneh FK Tolossa T Bekonjo NE Wakuma B. Time to recovery from severe acute malnutrition and its predictors among children aged 6–59 months at Asosa general hospital, Northwest Ethiopia. A retrospective follow up study. PLoS One. (2022) 17:e0272930. doi: 10.1371/journal.pone.0272930, PMID: 35960715 PMC9374216

[2] Girum T Muktar E Worku A. Comparative analysis of the survival status and treatment outcome of under-five children admitted with severe acute malnutrition among hospital-based and health center based stabilization centers, South Ethiopia. Open Public Health J. (2018) 11:209–20. doi: 10.2174/1874944501811010209

[3] World Food Programme. WFP global, operational response plan 2022. World Food Program. (2022). Available at: https://reliefweb.int/report/world/wfp-global-operational-response-plan-2022-update-7-february-2023 (Accessed on June 15, 2024).

[4] World food Programme. WFP global, operational response plan 2022. World Food Program. (2022). Available at: https://reliefweb.int/report/world/wfp-global-operational-response-plan-2024-update-10-february-2024 (Accessed on July 5, 2024).

[5] Joseph FI Falade A Earland J. Time to recovery and its predictors among children 6–59 months with acute malnutrition admitted to community inpatient therapeutic feeding centers in Katsina state, Northwest Nigeria: a retrospective review of health records (2010–2016). J Diarrhoeal Dis Res. (2023) 42:10–1. doi: 10.1186/s41043-023-00352-y, PMID: 36800992 PMC9936680

[6] Moyer JD Bohl DK Petry C Scott A Solórzano JR Kuhn R. The persistent global burden of severe acute malnutrition: cross-country estimates, models and forecasts. Glob Transit. (2020) 2:167–79. doi: 10.1016/j.glt.2020.07.004

[7] UNICEF. Level and trends in children malnutrition. (2020); Available at: https://www.who.int/publications/i/item/9789240003576 (Accessed on June 2024)

[8] Doku DT Neupane S. Double burden of malnutrition: increasing overweight and obesity and stall underweight trends among Ghanaian women. BMC Public Health. (2015) 15:1–9. doi: 10.1186/s12889-015-2033-6, PMID: 26178521 PMC4502461

[9] Ververs M Narra R. Treating cholera in severely malnourished children in the horn of Africa and Yemen. Lancet. (2017) 390:1945–6. doi: 10.1016/S0140-6736(17)32601-6, PMID: 28988791 PMC6262880

[10] UNICEF W. Joint child malnutrition estimates key findings. Unichef. (2020);16. Available at: https://data.unicef.org/resources/jme-report-2020/ (Accessed on June 11, 2024).

[11] Kuse KA Debeko DD. Spatial distribution and determinants of stunting, wasting and underweight in children under-five in Ethiopia. BMC Int Health Hum Rights. (2023) 23:641–17. doi: 10.1186/s12889-023-15488-z, PMID: 37016328 PMC10071774

[12] Bragança Lima MV Hinderaker SG Ogundipe OF Owiti PO Kadai B Maikere J. Association between cholera treatment outcome and nutritional status in children aged 2–4 years in Nigeria. PharmacoEconomics. (2021) 11:80–4. doi: 10.5588/pha.20.0078, PMID: 34159067 PMC8202622

[13] Belay E Handebo S Derso T Tariku A Sisay M. Prevalence and determinants of pre-adolescent (5-14 years) acute and chronic undernutrition Northwest Ethiopia. Int J Equity Health. (2019) 18:137. doi: 10.1186/s12939-019-1041-z 31477149 PMC6721279

[14] Adimasu M Sebsibie G Abebe F Baye G Abere K. Recovery time from severe acute malnutrition and associated factors among under-5 children in Yekatit 12 hospital, Addis Ababa, Ethiopia: a retrospective cohort study. Epidemiol Health. (2020) 42:1–11. doi: 10.4178/epih.e2020003 PMC7056942 32023778

[15] Atnafe B Roba KT Dingeta T. Time of recovery and associated factors of children with severe acute malnutrition treated at outpatient therapeutic feeding program in Dire Dawa, eastern Ethiopia. PLoS One. (2019) 14:1–16. doi: 10.1371/journal.pone.0217344 PMC6563956 31194757

[16] Belayneh M Loha E Lindtjørn B. Seasonal variation of household food insecurity and household dietary diversity on wasting and stunting among young children in a drought prone area in South Ethiopia: a cohort study. Culture Ecol Food Nutrit. (2021) 60:44–69. doi: 10.1080/03670244.2020.1789865, PMID: 32672490

[17] Müller O Krawinkel M. Malnutrition and health in developing countries. CMAJ. (2005) 173:279–86. doi: 10.1503/cmaj.050342, PMID: 16076825 PMC1180662

[18] Tadesse Z Teshome DF Lakew AM Debalkie G Gonete KA. Time to nutritional recovery and its determinants among children aged 6 to 59 months with severe acute malnutrition admitted to stabilization centers of WagHimra zone, Northeast Ethiopia. Ecol Food Nutr. (2021) 60:751–64. doi: 10.1080/03670244.2021.1907746, PMID: 33832358

[19] GTFCC. Cholera outbreak response: field manual. (2019); 26. Available at: http://www.who.int/cholera/publications/Cholera_outbreak_assessment.pdf (Accessed on September 2024)

[20] Gebrezgi D Teferi D Reddy PP. Recovery time from severe acute malnutrition and development of complementary food supplement for affected Ethiopian children. Int J Nutr. (2019) 3:1–6. doi: 10.14302/issn.2379-7835.ijn-19-2599

[21] Mamo WN Derso T Gelaye KA Akalu TY. Time to recovery and determinants of severe acute malnutrition among 6-59 months children treated at the outpatient therapeutic program in North Gondar zone, Northwest Ethiopia: a prospective follow up study. Ital J Pediatr. (2019) 45:136–8. doi: 10.1186/s13052-019-0732-9, PMID: 31684989 PMC6829982

[22] Tefera TK Abebe SM Hunegnaw MT Mekasha FG. Time to recovery and its predictors among children 6-59 months admitted with severe acute malnutrition to East Amhara hospitals, Northeast Ethiopia: a multicenter prospective cohort study. J Nutr Metab. (2020) 2020:1–8. doi: 10.1155/2020/5096201, PMID: 32963828 PMC7491447

[23] Kitesa GY Berhe TT Tedla GW Sahile AT Abegaz KH Shama AT. Time to recovery and its predictors among under five children in outpatient therapeutic feeding programme in Borena zone, southern Ethiopia: a retrospective cohort study. BMJ Open. (2023) 13:e077062–9. doi: 10.1136/bmjopen-2023-077062, PMID: 37709317 PMC10503381

[24] EPHI. Cholera hotspots in Ethiopia with OCV targets, February 18. (2020); Available at: http://Users/alexb/AppData/Local/MendeleyLtd/MendeleyDesktop/Downloaded/EPHI-2020-CholeraHotspotsinEthiopiawithOCVTargets.html (Accessed on May, 2022)

[25] Chowdhury MRK Rahman MS Billah B Rashid M Almroth M Kader M. Prevalence and factors associated with severe undernutrition among under-5 children in Bangladesh, Pakistan, and Nepal: a comparative study using multilevel analysis. ScientificReports. (2023) 13:1–12. doi: 10.1038/s41598-023-36048-w PMC10287716 37349482

[26] Sefako Makgatho Health Sciences University Mhlanga TPN Manafe M Sefako Makgatho Health Sciences University Ncube LJ Sefako Makgatho Health Sciences University. Feeding patterns and dietary diversity practices of caregivers with children (0 to 24 months) admitted with acute malnutrition in hospitals in Mpumalanga province, South Africa. Afr J Food Agric Nutr Dev. (2023) 23:24662–79. doi: 10.18697/ajfand.124.22735,

[27] EDHS CSA (CSA). Ethiopia demographic and health survey. (2016). Available at: https://dhsprogram.com/pubs/pdf/FR328/FR328.pdf (Accessed on May, 2023)

[28] Ethiopian Health and Nutrition Research Institute. National-cholera-guideline of Ethiopia. (2011). 123 p. Available at: http://repository.iifphc.org/handle/123456789/373 (Accessed on May, 2023)

[29] WHO. Guideline: updates on the management of severe acute malnutrition in infants and children Geneva: World Health Organization. (2013). Available at: https://iris.who.int/bitstream/handle/10665/95584/9789241506328_eng.pdf. (Accessed on June, 2024) 24649519

[30] FAO. *H. minimum* dietary diversity for women. A Guide for Measurement Rome: FAO. (2016). Available at: https://openknowledge.fao.org/server/api/core/bitstreams/088f944b-d268-4e04-b57d-027a3b6a56eb/content (Accessed on June,2024)

[31] Organization(FAO) F and agriculture. Undernourished people in the world. (2021). Available at: https://www.worldometers.info/undernourishment/ (Accessed on June, 2024)

[32] Federal Ministry of Health. National Guideline for the Management of Acute Malnutrition in Ethiopia. Natl Guidel Manag Acute Malnutrition Ethiop. (2019); 136. Available at: https://www.nutritioncluster.net/sites/nutritioncluster.com/files/2022-06/NationalGuidelineforManagementofAcuteMalnutritionMay%202019Version.pdf (Accessed on June, 2024)

[33] Sphere S. The sphere handbook Arabic. The Sphere Handbook Arabic (2018). Available at: https://spherestandards.org/wp-content/uploads/Sphere-Handbook-2018-EN.pdf (Accessed on June 2024)

[34] Gebremichael DY. Predictors of nutritional recovery time and survival status among children with severe acute malnutrition who have been managed in therapeutic feeding centers, southern Ethiopia: retrospective cohort study. BMC Int Health Hum Rights. (2015) 15:1–11. doi: 10.1186/s12889-015-2593-5, PMID: 26689192 PMC4687080

[35] Yadeta SK Tadesse T Negese T Haile B Kebede A Motuma A. Predictors of time to recovery from uncomplicated severe acute malnutrition among children in eastern Ethiopia. Front Nutr. (2024) 11:1–11. doi: 10.3389/fnut.2024.1275943, PMID: 38903630 PMC11187269

[36] Desyibelew HD Fekadu A Woldie H. Recovery rate and associated factors of children aged 6 to 59 months admitted with severe acute malnutrition at the inpatient unit of Bahir Dar Felege Hiwot referral hospital therapeutic feeding unite, Northwest Ethiopia. PLoS One. (2017) 12:1–12. doi: 10.1371/journal.pone.0171020, PMID: 28166247 PMC5293215

[37] Kidane GF Zereabruk K Aberhe W Hailay A Mebrahtom G Gebremeskel GG. Time to recovery from severe acute malnutrition and its predictors among under five children admitted to therapeutic feeding units of general and referral hospitals in Tigray, Ethiopia, 2020: a prospective cohort study. Pediatrics. (2023) 23:325–10. doi: 10.1186/s12887-023-04144-5, PMID: 37365604 PMC10291744

[38] Jarso H Workicho A Alemseged F. Survival status and predictors of mortality in severely malnourished children admitted to Jimma University specialized hospital from 2010 to 2012, Jimma, Ethiopia: a retrospective longitudinal study. Pediatrics. (2015) 15:1–13. doi: 10.1186/s12887-015-0398-4, PMID: 26174805 PMC4502938

[39] Fikrie A Alemayehu A Gebremedhin S. Treatment outcomes and factors affecting time-to-recovery from severe acute malnutrition in 6-59 months old children admitted to a stabilization center in southern Ethiopia: a retrospective cohort study. Ital J Pediatr. (2019) 45:46–9. doi: 10.1186/s13052-019-0642-x, PMID: 30971316 PMC6458656

[40] Baraki AG Akalu TY Wolde HF Takele WW Mamo WN Derseh B. Time to recovery from severe acute malnutrition and its predictors: a multicentre retrospective follow-up study in Amhara region, north-West Ethiopia. BMJ Open. (2020) 10:8–13. doi: 10.1136/bmjopen-2019-034583, PMID: 32060161 PMC7045195

[41] Ferdous F das SK Ahmed S Farzana FD Latham JR Chisti MJ. Severity of diarrhea and malnutrition among under five-year-old children in rural Bangladesh. Am J Trop Med Hyg. (2013) 89:223–8. doi: 10.4269/ajtmh.12-0743, PMID: 23817334 PMC3741240

[42] Altmann M Suarez-Bustamante M Soulier C Lesavre C Antoine C. First wave of the 2016-17 cholera outbreak in Hodeidah City, Yemen - ACF experience and lessons learned. Evidence for genomic applications. (2017) 9:1–15. doi: 10.1371/currents.outbreaks.5c338264469fa046ef013e48a71fb1c5, PMID: 29188130 PMC5693343

[43] Alam NH Islam S Sattar S Monira S Desjeux JF. Safety of rapid intravenous rehydration and comparative efficacy of 3 oral rehydration solutions in the treatment of severely malnourished children with dehydrating cholera. J Pediatr Gastroenterol Nutr. (2009) 48:318–27. doi: 10.1097/MPG.0b013e318180af27, PMID: 19274788

[44] Palmer DL Koster FT Alam AKMJ Islam MR. Nutritional status: a determinant of severity of diarrhea in patients with cholera. JINFECTDIS. (1976) 134:8–14. doi: 10.1093/infdis/134.1.8, PMID: 820813

[45] Vuppalapati C. Food security. Artificial Intelligence and Heuristics for Enhanced Food Security. (2022) 331:189–282. doi: 10.1007/978-3-031-08743-1_4

[46] Abitew DB Worku A Mulugeta A Bazzano AN. Rural children remain more at risk of acute malnutrition following exit from community-based management of acute malnutrition program in South Gondar zone, Amhara region, Ethiopia: a comparative cross-sectional study. PeerJ. (2020) 8:e8419. doi: 10.7717/peerj.8419, PMID: 32071802 PMC7008819

[47] Anato A. Severe acute malnutrition and associated factors among children under five years: A community-based-cross sectional study in Ethiopia. Heliyon. (2022) 8:1–6. doi: 10.1016/j.heliyon.2022.e10791, PMID: 36203897 PMC9529577

[48] Tegegne AS Belay DB. Predictors for time to recovery from sever acute malnutrition among under-five children admitted to therapeutic feeding unit at Dubti referral hospital, Afar region, Ethiopia. Pediatrics. (2021) 21:562–13. doi: 10.1186/s12887-021-03043-x, PMID: 34893039 PMC8662886

[49] Asiimwe JK Nambooze J Ssonko GW Kakande J Nyanzi L Kaddu P. Knowledge, attitudes and practices of caregivers of children 0 - 23 months in eastern and Central Uganda. FNS. (2021) 12:494–508. doi: 10.4236/fns.2021.126038

[50] Komugisa A Musoga RO. To asses the factors influencing the state of nutrition Care in the Inpatient Therapeutic Unit at Hoima Regional Referal Hospital in. (2023). Available at: https://sjhresearchafrica.org/index.php/public-html/article/download/371/254/1982 (Accessed on June 2024)

[51] Bogale SK Cherie N Bogale EK. Fathers involvement in child feeding and its associated factors among fathers having children aged 6 to 24 months in Antsokia Gemza Woreda, Ethiopia: cross-sectional study. PLoS One. (2022) 17:1–18. doi: 10.1371/journal.pone.0276565, PMID: 36441739 PMC9704664

[52] Paoli A Tinsley G Bianco A Moro T. The influence of meal frequency and timing on health in humans: the role of fasting. Nutrients. (2019) 11:1–19. doi: 10.3390/nu11040719, PMID: 30925707 PMC6520689

