# Supplementary material for: Determinants of recovery time from severe acute malnutrition among cholera-exposed and unexposed children in Ethiopia: a prospective cohort study
Source: Front Nutr. 2024 Oct 3;11:1463150. doi: 10.3389/fnut.2024.1463150 (PMC11484015; doi:10.3389/fnut.2024.1463150)
Supplement: Supplementary file 1 [file Data_Sheet_1.PDF]

Questionnaire

Part I. Socio-demographic Variables			
**S. no**	Questions	Response Options	Remark
101	code No.	_______________	
102	Name of kebele /numeration area	_______________	
103	Name of health facility	_______________	
104	Age of the child (years)	_______years	
105	Sex of the child	1. Male 0. Female	
106	Relation of the respondent to the child	Mother Caregiver<br>Father Other (Specify)____	
107	Mother's/caregiver's age	_________Years	
108	Place of residence of the /parent/ caregiver	1. Rural<br>0. Urban	
109	Occupation of Parent/caregiver	Farmer Housewife<br>Merchant, Daily laborer<br>Employee Other (specify)_________	
110`	Marital status of Parent/caregiver	1. Married 4. Separate<br>2. Single 5. Divorced<br>3. Widowed	
111	If married, the occupational status of your partner	Farmer Housewife<br>Merchant, Daily laborer<br>Employee Other (specify)___	
112	Religion of the Parent/caregiver	1. Orthodox 4. Catholic<br>2. Protestant 5. Others (specify)_____<br>3. Muslim	
113	Family size of the household	__________ Persons	
114	Number of children <15 years	__________ children	
115	Educational level of Parent/caregiver	Did not attend classes<br>Primary (1-8 grade)<br>Secondary (9-12)<br>Higher education (diploma and above)	
116	If married, the educational level of your partner.	Did not attend classes<br>Primary (1-8 grade)<br>Secondary (9-12)<br>Higher education (diploma and above)	
117	Partner involvement in child diet/feeding	Yes =1 No = 0	
118	Monthly income of the household	__________________in Birr	

Part II. Economic variables /Wealth status of the house. (EDHS 2016)			
**S. No**	Questions	Response Options	Remark
201.	Presence of own farmland land	(In Hec) ________	
202	The roof of the house with corrugated iron sheet	Yes =1 No = 0	
203	Type of the wall	1. Wooden and mud 3. Stone and cement<br>2. Stone and mud 4. Blocket<br>5. Bricks	

204	Type of house floor	1. Earth /Soil<br>2. Cement/Brick<br>3. Wooden/Bamboo<br>4. Ceramic<br>5. Other (Specify)	
205	How many rooms are used by the members of your household? (Excluding kitchen and store)	_____in number)	
206	Bank account	Yes =1 No = 0	
207	Mobile phone	Yes =1 No = 0	
208	Do you have the following kinds of livestock? If yes, the number of herds.	Number of cows/oxen___________<br>Number of horses / mules/donkeys_________<br>Number of goats/sheep __________<br>Number of chicken_____________<br>Other specify________________	
209	If you have livestock, where do they live?	In the same room with family<br>There is a separate room for themselves<br>Outside the room/'beret laye'	
210	Do you have the following items of furniture: (All or multiple Answers are possible)	Computer? Yes =1, No = 0<br>Tables Yes =1, No = 0<br>Chairs Yes =1, No = 0<br>Sofa Yes =1, No = 0<br>A bed with cotton/sponge/spring mattress? Yes =1, No = 0<br>Armoire Yes =1, No = 0<br>Cabinet Yes =1, No = 0<br>Other specify_______	
211	Does the family have a functional radio?	Yes = 1 No =0	
212	Does the family have a functional TV?	Yes = 1 No = 0	
213	Do you have the following items of furniture: (All or multiple Answers are possible)	Bicycle Yes =1, No = 0<br>Motorcycle/scooter Yes =1, No = 0<br>Animal-drawn cart Yes =1, No = 0<br>Car/truck Yes =1, No = 0<br>Boat with motor Yes =1, No = 0<br>Bajaj Yes =1, No = 0<br>Other specify_______<br>Washing machine Yes =1, No = 0<br>Microwave oven Yes =1, No = 0<br>Computer Yes =1, No = 0<br>Air conditioner Yes =1, No = 0	

Part III. Questions to assess household food security condition. (FANTA: 2007)

S. No	Questions	Response Options	Remark
301	In the past four weeks, did you worry that your household would not have enough food?	Yes =1<br>No = 0	If No go to →Q303
302	If yes Q301, for how often did this happen?	1. Rarely (1 or 2 times)	

		2. Sometimes (3 to 10 times)<br>3. Often (> 10 times)	
303	In the past four weeks, were you or any household member not able to eat the kinds of foods you preferred because of a lack of resources?	Yes =1<br>No = 0	If No go to →Q305
304	If yes Q303, how often did this happen?	1. Rarely (1 or 2 times)<br>2. Sometimes (3 to 10 times)<br>3. Often (> 10 times)	
305	In the past four weeks, did you or any household member have to eat a limited variety of foods due to a lack of resources?	Yes =1<br>No= 0	If No go to →Q308
306	If yes Q305, how often did this happen?	1. Rarely (1 or 2 times)<br>2. Sometimes (3 to 10 times)<br>3. Often (> 10 times)	
307	In the past four weeks, did you or any household member have to eat some foods that you did not want to eat because of a lack of resources to obtain other types of food?	Yes =1<br>No = 0	If No go to →Q309
308	If yes Q307, how often did this happen?	1. Rarely (1 or 2 times)<br>2. Sometimes (3 to 10 times)<br>3. Often (> 10 times)	
309	In the past four weeks, did you or any household member have to eat a smaller meal than you felt you needed because there was not enough food?	Yes =1<br>No= 0	If No go to →Q311
310	If yes Q309, how often did this happen?	1. Rarely (1 or 2 times)<br>2. Sometimes (3 to 10 times)<br>3. Often (> 10 times)	
311	In the past four weeks, did you or any other household member have to eat fewer meals in a day because there was not enough food?	Yes =1<br>No= 0	If No go to →Q313
312	If yes Q311, how often did this happen?	1. Rarely (1 or 2 times)<br>2. Sometimes (3 to 10 times)<br>3. Often (> 10 times)	
313	In the past four weeks, was there ever no food to eat of any kind in your household because of lack of resources to get food?	Yes =1<br>No = 0	If No go to →Q315
314	If yes Q313, how often did this happen?	1. Rarely (1 or 2 times)<br>2. Sometimes (3 to 10 times)<br>3. Often (> 10 times)	
315	In the past four weeks, did you or any household member go to sleep at night hungry because there was not enough food?	Yes =1<br>No = 0	If No go to →Q317
316	If yes Q315, how often did this happen?	1. Rarely (1 or 2 times)<br>2. Sometimes (3 to 10 times)<br>3. Often (> 10 times)	
317	In the past four weeks, did you or any household member go a whole day and night without eating anything because there was not enough food?	Yes =1<br>No = 0	
318	If yes Q317, how often did this happen?	1. Rarely (1 or 2 times)<br>2. Sometimes (3 to 10 times)<br>3. Often (> 10 times)	

Part IV: Water, sanitation, and hygiene (WASH). (UNCH, WHO 2022)			
**Water source and use, Sanitation and hygiene**			
S. No	Questions	Response Options	Remark
401	Do you have a separate room, which is used, as a Kitchen?	Yes= 1<br>No = 0	
402			
403	What is your main source of drinking water?	1. Piped into a dwelling<br>2. Public tap<br>3. Other Improved water supply (Protected spring & well)<br>4. Unimproved (Unprotected spring & well, river/stream, Dam)	
404	How much time is required to fetch water from the house to the water source?	Piped into a dwelling<br>1-15 minutes<br>16-30 minutes<br>30 - 60 minutes<br>60 minutes above	
405	Average household water use per day per person for drinking, cooking, and personal hygiene	In liters____________	
405	How could you draw water from the storage?	Pouring, 2. Deeping<br>3. Others (specify) ________	
406	If the Q405 is Deeping, do you use a separate cap for drawing water from the water storage container?	Yes = 1<br>No = 0	
407	Do you treat water in any way to make it safer at home?	Yes = 1<br>No = 0	
408	If the answer to Q 407 is yes, what activities do you practice?	1. Boiling<br>2. Chemical treatments<br>3. Filtering using cloth<br>4. filtering using sand, gravel or ceramics<br>5. Sun radiation treatment<br>6. Sedimentation<br>7. Others (specify) _____________	
409	Does the drinking water storage container have a cover?	Yes = 1<br>No = 0	
410	How is the cleanness of the water storage container (inside and outside?	Very clean<br>Presence of some filth material<br>It is dirty?	
411	Is the water storage container put above 40 cm on the floor?	Yes = 1<br>No = 0	
412	How frequently clean storage container material	Always before fetching water<br>Daily<br>Weekly<br>If the material looks dirty	
413	Do you wash your hands before fetching water?	Yes = 1<br>No = 0	
414	Do you have latrine?	Yes = 1 No = 0	
415	If the answer to Q 413 is yes, what type of latrine do the households use?	1. Private traditional pit latrine<br>2. Private wooden slab latrine	

		3. Private cement slab latrine<br>4.Private VIP latrine<br>5. Shared wooden slab latrine<br>6. Shared VIP latrine<br>7. Flash to the sewerage system<br>9. Other (Specify)_______________	
416	If yes Q414, is it in use?<br>*feces are not seen around the latrine	Yes = 1<br>No = 0	
417	How often is the latrine cleaned?	Every day<br>1-2 times per week<br>1-2 times per month<br>Not cleaned	
418	If yes Q416, how is the cleanliness / hygienic condition of the latrine?	Clean =1<br>Not clean = 0	
419	If the answer for Q 416 is no, where do the households defecate?	1. Designated area<br>2. Bush<br>3. At the back yard<br>4. Open field<br>5. Others (specify)____	
420	Are your children able to use the latrine on their own?	Yes = 1<br>No = 0	
421	If yes for Q420, how do you care after using the latrine?	With water<br>With soft tissue/paper<br>Not clean at all<br>Other, specify____	
422	If no for Q420, where do they defecate?	Disposing into latrine<br>Bring in the pit<br>Disposing at the drainage<br>Put into solid waste<br>Dispose at the housing compound	
423	Where do you dispose of wastewater?	Sewage system<br>Garde<br>Pond<br>Other:___	
424	Do you use the stool as fertilizer	Yes =1 No = 0	
425	Distance between toilet and water source	0 – 5 meters<br>6 – 10 meters<br>11– 20 meters<br>21–29 meters<br>30 meters or more	
426	Mainly, how does the household dispose of solid wastes?	In a private pit<br>Common pit<br>Composting<br>Buried<br>Burning<br>Open field disposal<br>Other (specify)_	
427	Is there a hand-washing facility near a latrine?	Yes = 1<br>No = 0	
428	If yes for Q 427, is there water in the container?	Yes = 1<br>No = 0	
429	If yes for Q428, is there moisture observed in the ground?	Yes = 1<br>No = 0	
430	If yes for Q433, at the hand washing facility?	Is there soap? Yes = 1, No = 0<br>Is there a soap container? Yes = 1, No = 0	
431	Does food preparation utensil have their own rack/stand?	Yes = 1<br>No = 0	
432	How is the cleanliness of the rack?	Yes Clean = 1<br>No dirty materials are there = 0	

433	When do you need to wash your hands? All can apply	After going to the toilet/latrine<br>After cleaning a baby's bottom/changing a baby's nappy<br>Before preparing/handling food<br>Before feeding a child/eating<br>After handling raw food<br>After handling garbage<br>Other<br>Don't know	

Part V: practice, Attitude & knowledge of malnutrition. (EDHS: 2016; Ghana FTF Baseline Survey 2012)

Section one: child feeding practice (household Nutrition)

S.No	Questions	Response Options	Remark
501.	How many times does your child feed in a day?	___________times	
502	Is there a time that your child refuses to eat food?	Yes = 1<br>No = 0	If No go to → Q504
503	If yes in Q502, what do you do in such a situation? Example: is it due to appetite loss?	Leave him/her alone or stop feeding<br>Forced feeding<br>Give small feeds<br>Change feeds<br>Sought medical help<br>Other(specify)	
504	Are there foods that your patient (child) likes/tolerates?	Yes = 1<br>No = 0	
505	If yes in Q502, what are they?	__________________	

Section two: attitude questions about nutrition

S.No	Questions	Response Options	Remark
506	Some mothers believe that when children become undernourished, that is they stop growing or lose weight.	1. Agree<br>2. Neural 3. Disagree	
507	According to their feeling of some mothers diversified food improves child health	1. Agree<br>2. Neural 3. Disagree	
508	Some mothers believe that the food a child eats keeps him/her nutritious	1. Agree<br>2. Neural 3. Disagree	
509	Some mothers believe that the food a child eats should contain vitamins and minerals	1. Agree<br>2. Neural 3. Disagree	
510	It is believed that giving a healthy and balanced diet to children is important	1. Agree<br>2. Neural 3. Disagree	
511	According to some mothers' feeling children should eat what they like no need to worry about the healthiness of food	1. Agree<br>2. Neural 3. Disagree	

Section three: knowledge questions about malnutrition

512	Have you ever received information (education/training) on nutrition?	Yes = 1<br>No = 0	
513	Did you receive counseling services about child feeding from health professionals?	Yes = 1<br>No = 0	

514	What are the Signs and symptoms of malnourished children?<br>(*can answer more than one if applicable)	Lack of energy/weakness:<br>Weaker immune system<br>Loss of weight/thinness<br>Children do not grow as they should (growth faltering)<br>Don't know<br>Other________	
515	Have you heard about a balanced diet?	Yes = 1, No = 0	
516	If yes mention	Feeding Infant/Child a variety of food and a balanced diet<br>Feeding Infant/Child a lot of food<br>Feeding infant/child a lot of meat<br>Other (Specify) ____________	
517	What are the consequences of an unbalanced deity?<br><br>(*can answer more than one if applicable)	Poor health conditions/ illness<br>Weaker immune system<br>Loss of weight/thinness<br>Death<br>Do not know<br>Others (Specify) __________	
518	What can be done to improve a child's nutrition/ to prevent undernutrition among children?<br>(*can answer more than one if applicable)	Feed frequently<br>Feed the child well (balanced/varied diet)<br>Improve sanitation<br>Do not know<br>Other (specify)__________	
519	Which types of food should not be given to the child? (Food taboos)	_______________	
520	Do you give special food to your child during illness?	Yes = 1, No = 0	
521	Do you withhold food from your child when ill?	Yes = 1, No = 0	
522	Do you give special food to your child after illness?	Yes = 1, No = 0	
523	What is the amount of fluid you give to your child during diarrhea?	The same<br>Less than Usual<br>More than usual	

Part VI: Food frequency questionnaire (FFQ) for <15 years children. (FAO and FHI 360. 2016)

Thinking back on the last month, please tell me how often you consumed each of the following items. You can tell me in terms of times per day, per week, or month. Enter 0 if never consumed the item, and enter the number of meals if consumed the item

K5. Teff	Per day		K6. Maize	Per day	
	Per week			Per week	
	Per month			Per month	
K7. Barley	Per day		K8. Wheat, including bread	Per day	
	Per week			Per week	
	Per month			Per month	

K9. Sorghum /millet	Per day<br>Per week<br>Per month		K10. Rice	Per day<br>Per week<br>Per month	
In the last three months, how often did you consume each of the following?					
K11. Beef	Per day<br>Per week<br>Per month		K12. Pork	Per day<br>Per week<br>Per month	
K13. Fish	Per day<br>Per week<br>Per month		K14. Chicken	Per day<br>Per week<br>Per month	
K15. Goat/Lamb	Per day<br>Per week<br>Per month		K16. Liver	Per day<br>Per week<br>Per month	
K17. Milk	Per day<br>Per week<br>Per month		K18. Cheese	Per day<br>Per week<br>Per month	
K19. Butter	Per day<br>Per week<br>Per month		K20. Eggs	Per day<br>Per week<br>Per month	
K21. Nuts	Per day<br>Per week<br>Per month		K22. Oil	Per day<br>Per week Per month	
K23. Beans, peas, lentils	Per day<br>Per week<br>Per month		K24. Sweet potatoes	Per day<br>Per week Per month	
K25. Potatoes	Per day<br>Per week<br>Per month		K26. Carrot	Per day<br>Per week Per month	
K27. Tomato	Per day<br>Per week<br>Per month		K28. Cauliflower	Per day<br>Per week<br>Per month	
K29. Leafy green vegetables	Per day<br>Per week<br>Per month		K30. Avocado	Per day<br>Per week<br>Per month	
K31. Papaya	Per day		K32. Bananas	Per day	

	Per week			Per week	
	Per month			Per month	
K33. Pineapple	Per day		K34. Oranges	Per day	
	Per week			Per week	
	Per month			Per month	
K35. Coffee	Per day		K36. Soft drinks	Per day	
	Per week			Per week	
	Per month			Per month	
K37. Onion	Per day		K38. Strawberry	Per day	
	Per week			Per week	
	Per month			Per month	
K39. Other specify	Per day		K40. Other specify	Per day	
	Per week			Per week	
	Per month			Per month	

Part VII: Clinical condition of the children.			
**S. No**	Questions	Response Options	Remark
701	Was the child exposed to cholera? (Exposure status of children to cholera)	Yes = 1<br>No = 0	
702	If yes when the patient had recovered from cholera	___/___/_____(Date/Month/Year)	
703	Date of follow-up start for malnutrition	____/___/_____(Date/Month/Year)	
704	Does the child have edema?	Yes =1 No = 0	
705	Did the child have a history of diarrhea, signs/symptoms of SAM in the last two weeks	Yes =1 No = 0	
706	Did the child have a history of co-morbid illness in the last two weeks?	Yes =1 No = 0	
709	Cholera Vaccine status	Fully Vaccinated<br>Not fully Vaccinated	
**Part VIII: Anthropometric measurements of the child <15years, to the nearest 0.1kg and 0.1cm)**			
M1.	Weight	______KG	
M2.	Height	______CM	
M3.	MUAC	______CM	

// I have finished my questions, thank you for your time and information.

I wish you to stay safe and healthy //
