# Supplementary material for: Determinants of recovery time from severe acute malnutrition among cholera-exposed and unexposed children in Ethiopia: a prospective cohort study
Source: Front Nutr. 2024 Oct 3;11:1463150. doi: 10.3389/fnut.2024.1463150 (PMC11484015; doi:10.3389/fnut.2024.1463150)
Supplement: Supplementary file 2 [file Data_Sheet_2.PDF]

Operational definitions

Mother/caregiver	Parent or guardian of the children with Severe Acute Malnutrition (SAM)
Severe Acute Malnutrition (SAM)	Description of malnutrition level encompassing children 6-59 months with < -3 z-scores, and/or MUAC < 11.5 cm, and /or bilateral pitting nutritional edema, persons with SAM have higher morbidity and mortality risks. (SAM guideline, Ethiopia 2019)
Food insecurity	Lack of regular access to enough safe and nutritious food for normal growth and development and an active and healthy life. (FAO 2024)
Dietary Diversity	Dietary diversity is a qualitative measure of food consumption that reflects household access to a variety of foods and is also a proxy for the nutrient adequacy of individuals' diets. (FAO 2024)
Attitude of mother/caregiver on children's dietary habits	Good attitude: The mother/caregiver answered the attitude questions about children's dietary habits average and above<br><br>Poor attitude: Mother/caregiver answered the attitude questions about children's below-average dietary habits.
Knowledge of mother/caregiver on children's dietary habit	Good knowledge: Mother/caregiver answered the knowledge questions about children's dietary habits average and above<br><br>Poor knowledge: The mother/caregiver answered the knowledge questions about children's dietary habits below average
Time to recover	It is the time duration that SAM children take to recover from their SAM.
Recovered	When the MUAC measurement of children is >11.5 cm, we say children have recovered from their SAM.
